# Phenome-wide analysis highlights putative causal relationships between self-reported migraine and other complex traits

**DOI:** 10.1186/s10194-021-01284-w

**Published:** 2021-07-08

**Authors:** Luis M. García-Marín, Adrián I. Campos, Nicholas G. Martin, Gabriel Cuéllar-Partida, Miguel E. Rentería

**Affiliations:** 1 Department of Genetics and Computational Biology, QIMR Berghofer Medical Research Institute, Brisbane, QLD Australia; 2 School of Biomedical Sciences, Faculty of Medicine, The University of Queensland, Brisbane, QLD Australia; 3 The University of Queensland Diamantina Institute, The University of Queensland, Woolloongabba, QLD Australia; 4 Present address: 23andMe, Inc, Sunnyvale, California USA

**Keywords:** Migraine, Genetics, Causal inference, Epidemiology, Complex traits

## Abstract

**Background:**

Migraine is a complex neurological disorder that is considered the most common disabling brain disorder affecting 14 % of people worldwide. The present study sought to infer potential causal relationships between self-reported migraine and other complex traits, using genetic data and a hypothesis-free approach.

**Methods:**

We leveraged available summary statistics from genome-wide association studies (GWAS) of 1,504 phenotypes and self-reported migraine and inferred pair-wise causal relationships using the latent causal variable (LCV) method.

**Results:**

We identify 18 potential causal relationships between self-reported migraine and other complex traits. Hypertension and blood clot formations were causally associated with an increased migraine risk, possibly through vasoconstriction and platelet clumping. We observed that sources of abdominal pain and discomfort might influence a higher risk for migraine. Moreover, occupational and environmental factors such as working with paints, thinner or glues, and being exposed to diesel exhaust were causally associated with higher migraine risk. Psychiatric-related phenotypes, including stressful life events, increased migraine risk. In contrast, *ever feeling unenthusiastic / disinterested for a whole week*, a phenotype related to the psychological well-being of individuals, was a potential outcome of migraine.

**Conclusions:**

Overall, our results suggest a potential vascular component to migraine, highlighting the role of vasoconstriction and platelet clumping. Stressful life events and occupational variables potentially influence a higher migraine risk. Additionally, a migraine could impact the psychological well-being of individuals. Our findings provide novel testable hypotheses for future studies that may inform the design of new interventions to prevent or reduce migraine risk and recurrence.

**Supplementary Information:**

The online version contains supplementary material available at 10.1186/s10194-021-01284-w.

## Introduction

Migraine is a complex neurological disorder characterised by intense, debilitating headaches on one side of the head, and it is often accompanied by nausea, vomiting, numbness, and sensitivity to light and sound. Migraine is the most common disabling brain disorder, affecting 14 % of the population [1–5].

A migraine episode is commonly composed of three stages [1, 3, 6, 7]. First, during the prodromal phase, which can last from a couple of hours to several days, individuals may experience fatigue, lack of concentration, excessive yawning, irritability, neck stiffness, light sensitivity, and nausea [1, 3, 6, 7]. Then, in the headache phase, which can last a few hours or days, individuals are affected by strong headaches on one side of the head along with dizziness, light sensitivity, and vomiting [1, 3, 6, 7]. Finally, the postdromal phase lasts a few hours or a day, and individuals are no longer afflicted by headache pain. Still, individuals can experience fatigue, irritability, nausea, light sensitivity, and lack of concentration [1, 3, 6, 7].

Migraine is a heritable trait, with heritability estimated between 30 and 60 % [8]. Genome-wide association studies (GWAS) have led to substantial advances in understanding migraine’s genetic aetiology. In particular, GWAS have identified over 40 genomic risk loci for migraine [9] and genetic overlap between migraine and other phenotypes, including attention-deficit hyperactive activity disorder (ADHD), major depressive disorder, epilepsy, stroke, and coronary artery disease [8]. Nonetheless, the identification of a shared genetic architecture between traits does not imply a causal relationship. A genetic correlation could be explained by vertical pleiotropic effects (i.e., a genetic variant’s effect on a trait is mediated by its effect on another trait) or by horizontal pleiotropy (i.e., genetic variants have an effect on both traits). In particular, horizontal pleiotropy, and sample overlap, are known to bias estimates and increase the possibility of false-positive findings in genetic epidemiology studies using traditional methods, such as Mendelian randomisation, to assess causality [10, 11].

As an alternative to Mendelian randomisation, the latent causal variable method (LCV) was developed to provide an opportunity to investigate potential causal associations with a different approach (see Methods) [10]. Advantages of the LCV method include that it is able to minimise the effects of sample overlap [10, 12–14], it is less susceptible to confounding by horizontal pleiotropy [10, 12], and it uses aggregated genetic information throughout the entire genome to increase statistical power, allowing to test “underpowered” phenotypes with none or few significant genome-wide loci [10, 12–14].

Despite extensive efforts to understand migraine’s aetiology, there is still a growing need to generate investigation lines that can successfully incorporate results and hypotheses from genetic studies of migraine into clinical practice. That could enable the identification of disease-modifying traits and pinpoint the underlying causes of this complex neurological disorder. Here, we conduct a multi-trait analysis of GWAS with samples for self-reported migraine in the 23andMe cohort and chronic headaches in the UK Biobank. Then, we leverage an extensive collection (*N* = 1,504) of GWAS summary statistics to conduct a hypothesis-free phenome-wide screening of traits causally associated with migraine using the latent causal variable (LCV) method. Our results generate testable hypotheses for future studies and provide novel insights into the relationship between migraine and overall health.

## Methods

### Sample

The present study used samples from the 23andMe and UK Biobank cohorts. Details are given below.

#### 23andMe cohort

The 23andMe cohort comprised 30,465 self-reported migraine cases and 143,147 controls and was previously included in the meta-analysis by Gormely et al. [15]. Participants provided informed consent and reported their migraine diagnosis. Covariates included sex, age, and five principal genetic components.

#### UK Biobank cohort

We used information for chronic headaches as a proxy trait for migraine, as several cases of migraine might have been undiagnosed or lacking an ICD10 code. Chronic headaches were defined as pain in the head lasting for at least three months. In total, 39,283 (~ 9 %) of the UK Biobank participants included in the analysis were considered cases for chronic headaches.

Chronic headache was defined using the item “Have you had headaches for more than three months?” (Field-ID: 3799), which could be answered with “Yes”, “No”, “Do not know”, or “Prefer not to answer”. Individuals that selected “Yes” were defined as cases, whereas those who selected “No” for headaches for more than three months were defined as controls. GWAS was performed using REGENIE (v1.0.6.2), a method that implements a logistic mixed-effects model. This approach models genetic relationships between individuals as a random effect to account for cryptic relatedness. Quality control consisted of excluding variants with minor allele frequency (MAF) < 0.005, imputation quality < 0.6 and those deviating from Hardy–Weinberg equilibrium (*p*-value < 1 × 10^− 5^). After quality control exclusions, a total of 11,172,285 single nucleotide polymorphisms (SNPs) remained in the analysis. Subject data were excluded if their genotype-derived principal components 1 and 2 were further than six standard deviations away from 1000 Genomes European sample population. Sex, age, genotyping array, and the top 10 principal components derived from genetic data were used as covariates for the association analysis. After quality control, a total of up to 441,088 individuals remained in the GWAS.

### GWAS Multivariate analysis

Given that chronic headaches and self-reported migraine represent highly related but not the same trait, we performed a multi-trait analysis of GWAS (MTAG) analysis [16]. This approach leverages LD-score regression [17] to estimate a genetic correlation between two GWAS and perform a weighted meta-analysis and boost statistical power. We used MTAG(v2019/08/26) to combine the 23andMe self-reported migraine GWAS summary statistics with the UK-Biobank chronic headaches GWAS and observed a robust genetic correlation (r_G_=0.83 s.e.=0.05). Because the current study’s scope is migraine, we focused on the output specific to migraine, which had > 35 genome-wide significant loci.

### Datasets

The Complex Traits Genomics Virtual Lab (CTG-VL; https://genoma.io/) [18] has made available a compilation of GWAS summary statistics for 1,504 traits. A substantial proportion of these correspond to the second wave of GWAS results from the UK Biobank released by the Neale Lab (www.nealelab.is/uk-biobank/) [19], and the rest come from multiple GWAS consortia. Thus, most GWAS were performed using European ancestry individuals, and traits include objective laboratory measurements, self-reported phenotypes, and consortia meta-analyses. UK Biobank GWAS were adjusted for age, age-squared, inferred sex, age * inferred sex, age-squared * inferred sex, and 20 genetic ancestry principal components [18, 19]. For the present study, only GWAS derived from European populations were used to avoid biases due to population differences in linkage-disequilibrium and allele frequencies [18].

### Genetic correlations

A genetic correlation indicates the extent to which genetic effect sizes at common genetic variants are shared between two different phenotypes [13, 20]. We performed genetic correlation analyses using an offline version of CTG-VL for the LCV method, in which a linkage disequilibrium score regression [17] is used to assess a genetic correlation between two traits. Multiple testing was corrected for using Benjamini-Hochberg’s False Discovery Rate (FDR < 5 %).

### Genetic causal proportion

We estimated the genetic causal proportion (GCP) between migraine and 1,504 other phenotypes using the phenome-wide analysis pipeline in the CTG-VL as described in previous studies [12–14] to assess if a significant genetic correlation could be explained by a potential causal relationship between the two traits. Shortly, the CTG-VL implemented the phenome-wide LCV analysis pipeline in R 4.0.0 [12] based on the R script that the original authors of the LCV method [10] have made available in the Github repository (https://github.com/lukejoconnor/LCV). Further, to ensure consistency of alleles and variants across GWAS summary statistics, data was formatted using munge_sumstats.py, which is available by the LD-score software and extracted hapmap SNPs using the provided list of SNPs (w_hm3.snplist) (https://github.com/bulik/ldsc/wiki). In the present study, we uploaded GWAS summary statistics for migraine onto CTG-VL. Then, we performed the phenome-wide analysis pipeline to estimate genetic correlations and GCP estimates with LD-score regression and LCV, respectively. Finally, we used causal architecture plots to illustrate our results. A detailed and illustrated description of this approach is available in previous studies [12, 13].

Regarding correction for multiple comparisons, we applied the LCV method to estimate the GCP for all phenotypes with evidence of a genetic correlation with self-reported migraine at Benjamini-Hochberg’s False Discovery Rate (FDR < 5 %). Then, FDR < 5 % was used to account for multiple comparisons and identify those traits that had evidence of a potential causal relationship based on their GCP.

LCV leverages GWAS summary statistics to estimate a genetic correlation and relies on a latent variable *L*, assumed to be the causal constituent mediating the genetic correlation between both traits, to estimate the genetic causality proportion (GCP) [10, 12, 13, 21]. An absolute GCP value of 1 indicates the detection of a causal association that could be explained by vertical pleiotropic effects among a pair of genetically correlated phenotypes (i.e., the effect of a genetic variant on a trait is mediated by its effect on another trait). In contrast, a GCP value of zero implies horizontal pleiotropic effects between the phenotypes, in which case an intervention on any of them would not affect the other, a consequence of the absence of genetic causality between them [10, 12, 13]. Also, a |GCP| < 0.60 is considered low and indicates limited partial genetic causality [10]. Multiple testing was corrected for using Benjamini-Hochberg’s False Discovery Rate (FDR < 5 %).

## Results

We identified 510 significant genetic correlations of which 17 were found to be potential causal associations with self-reported migraine (|GCP| > 0.60; FDR < 5 %; Supplementary File 1) and one showed evidence of limited partial genetic causality (|GCP| < 0.60; FDR < 5 %; Supplementary File 1).

**Fig. 1:**
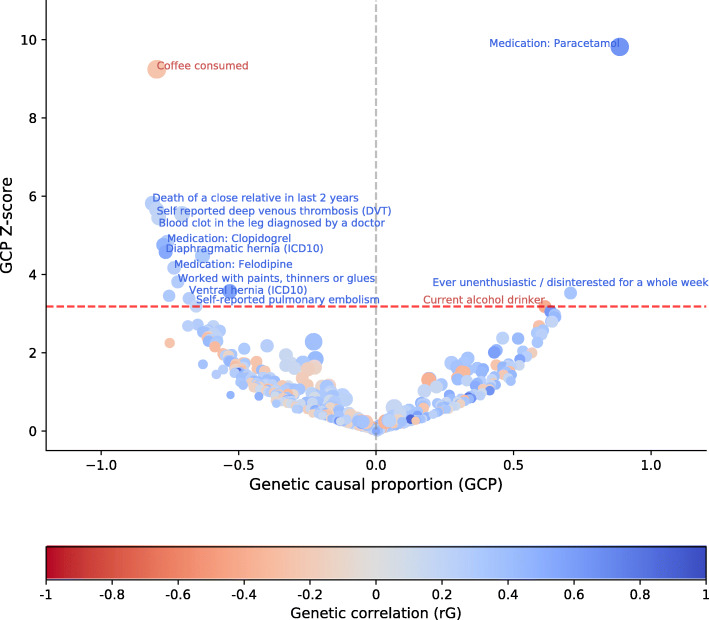
Potentially causal associations for migraine. Causal architecture plots illustrating the latent causal variable exposome-wide analysis results. Each dot represents a trait with a significant genetic correlation with migraine. The y-axis shows the genetic causality proportion (GCP) absolute Z-score (statistical significance), whilst the x-axis exhibits the GCP estimate. The red dashed lines represent the statistical significance threshold (FDR < 5 %), while the division for traits causally influencing migraine (on the left ) and traits causally influenced by migraine (on the right) is represented by the grey dashed lines. Phenotypes in blue show a positive genetic correlation with migraine, while phenotypes in red show a negative genetic correlation with migraine

Traits identified to potentially increase self-reported migraine risk include phenotypes allocated in the International Classification of Diseases (ICD10) such as *diaphragmatic hernia* (r_G_ = 0.53, GCP = -0.77, GCP_pvalue_ = 4.85 × 10 ^− 06^), *ventral hernia* (r_G_ = 0.27, GCP = -0.68, GCP_pvalue_ = 7.04 × 10 ^− 04^), and *benign neoplasm of colon, rectum, anus and anal canal* (r_G_ = 0.19, GCP = -0.71, GCP_pvalue_ = 2.91 × 10 ^− 08^) (Fig. 1; Table 1).

**Table 1:** Traits with an inferred causal relationship with migraine

Trait	GCP	GCP se	GCP pval	r_G_	r_G_ se	r_G_ pval
Medication: Paracetamol	0.89	0.09	9.70E-23	0.66	0.03	2.75E-90
Coffee consumed	-0.80	0.09	2.40E-20	-0.25	0.10	1.36E-02
Death of a close relative in last 2 years	-0.81	0.14	6.00E-09	0.27	0.06	3.55E-05
Self reported deep venous thrombosis (DVT)	-0.80	0.14	1.59E-08	0.21	0.07	2.61E-03
Benign neoplasm of colon, rectum, anus and anal canal (ICD10)	-0.71	0.13	2.91E-08	0.19	0.05	5.30E-04
Blood clot in the leg diagnosed by a doctor	-0.79	0.15	5.15E-08	0.19	0.07	6.22E-03
Medication: Clopidogrel	-0.76	0.16	1.38E-06	0.27	0.11	1.48E-02
Medication: Dihydrocodeine	-0.77	0.16	1.98E-06	0.47	0.16	3.75E-03
Diaphragmatic hernia (ICD10)	-0.77	0.17	4.85E-06	0.53	0.08	6.45E-11
Stroke diagnosed by doctor	-0.63	0.14	7.55E-06	0.32	0.11	3.83E-03
Medication: Felodipine	-0.73	0.18	3.06E-05	0.27	0.09	2.03E-03
Worked with paints, thinners or glues	-0.72	0.19	1.39E-04	0.28	0.09	1.71E-03
Ever unenthusiastic / disinterested for a whole week	0.71	0.20	4.25E-04	0.34	0.05	8.12E-11
Workplace sometimes had a lot of diesel exhaust	-0.75	0.22	5.55E-04	0.31	0.08	2.44E-04
Ventral hernia (ICD10)	-0.68	0.20	7.04E-04	0.27	0.10	8.35E-03
Self-reported pulmonary embolism	-0.65	0.21	1.45E-03	0.19	0.07	1.13E-02
Current alcohol drinker	0.61	0.19	1.47E-03	-0.39	0.04	1.51E-19

This table shows all traits with a significant (FDR < 5 %) and strong genetic causal proportion (|GCP| > 0.60) with migraine. Due to space constraints, results for all nominally significant genetic correlations for migraine are shown in Supplementary File 1.

*Trait *Trait causally associated with obesity, *GCP *Genetic causal proportion, *GCP se *Genetic causal proportion standard deviation, *GCP pval *Genetic causal proportion unadjusted *p*-value, *r*_*G*_ Genetic correlation, *r*_*G*_* se* Genetic correlation standard deviation, *r*_*G*_* p val* Genetic correlation unadjusted *p*-value

Our results show that self-reported migraine influences an increase in the intake of the analgesic *Paracetamol* (r_G_ = 0.66, GCP = 0.77, GCP_pvalue_ = 9.70 × 10 ^− 23^). In contrast, a higher self-reported migraine risk is influenced by traits involving the use of various medications such as, *Clopidogrel*, which is an antiplatelet drug, and *Felodipine* commonly prescribed for hypertension (Fig. 1; Table 1).

Self-reported cardiovascular phenotypes such as *deep venous thrombosis (DVT)* (r_G_ = 0.21, GCP = -0.80, GCP_pvalue_ = 1.59 × 10 ^− 08^) and *pulmonary embolism* (r_G_ = 0.19, GCP = -0.65, GCP_pvalue_ = 1.45 × 10 ^− 03^) appear to increase self-reported migraine risk, as do some phenotypes diagnosed by a doctor, including a *blood clot in leg* (r_G_ = 0.19, GCP = -0.79, GCP_pvalue_ = 5.15 × 10 ^− 08^) and *stroke* (r_G_ = 0.32, GCP = -0.63, GCP_pvalue_ = 7.55 × 10 ^− 06^) (Fig. 1; Table 1).

For psychiatric-related phenotypes, self-reported migraine was a potential outcome of stressful life events such as *death of a close relative in the last two years* (r_G_ = 0.27, GCP = -0.81, GCP_pvalue_ = 6.00 × 10 ^− 09^), whilst *ever feeling unenthusiastic / disinterested for a whole week* (r_G_ = 0.53, GCP = 0.70, GCP_pvalue_ = 4.25 × 10 ^− 04^) was a putative migraine consequence.

Occupational-related variables such as *worked with paint, thinner or glues* (r_G_ = 0.28, GCP = -0.72, GCP_pvalue_ = 1.39 × 10 ^− 04^) and being at a *workplace that had a lot of diesel exhaust* (r_G_ = 0.25, GCP = -0.81, GCP_pvalue_ = 3.69 × 10 ^− 04^) potentially increase self-reported migraine risk (Fig. 1; Table 1).

## Discussion

The present study contributes to advance the understanding of migraine’s aetiology by providing insights into migraine’s causal architecture. We used GWAS data to investigate potential causal associations between self-reported migraine and 1,504 different phenotypes and identified 18 inferred causal associations. Overall, our results show a putative vascular component to self-reported migraine and suggest that abdominal discomfort and stressful life events could increase self-reported migraine risk.

Abdominal discomfort has been previously associated with migraine [22–24]. For example, it has been shown that migraine among patients with abdominal discomfort can be eased once the abdominal pain source is treated [22, 23]. Thus, a plethora of studies seeks to determine whether gastrointestinal factors could influence the development of a migraine episode. However, the range of gastrointestinal abnormalities and disorders related to migraine has not been entirely disclosed. In the present study, phenotypes such as diaphragmatic hernia (ICD10), ventral hernia (ICD10), and benign neoplasm of colon, rectum, anus and anal canal (ICD10), which are known to be usually accompanied by inflammation and abdominal pain or discomfort [25–28], posed a putative causal effect increasing self-reported migraine risk. Therefore, our results are aligned with the hypothesis in which abdominal pain could be a risk factor for migraine, possibly through inflammatory or vascular mediators [23]. These findings could be used as testable hypotheses in future studies, which in turn should seek to describe the possible molecular underpinnings underlying the relationship between migraine and sources of abdominal pain or discomfort.

Previous research has aimed to describe the extent to which vascular components explain migraine [29–31]. Mendelian randomisation studies have identified diastolic blood pressure as potentially causal for migraine [32]. In our study, self-reports of *pulmonary embolism* and *deep venous thrombosis* and traits diagnosed by a doctor such as a *blood clot in the leg* increased self-reported migraine risk. Similarly, the use of medications for cardiovascular disorders was also found to increase the risk for self-reported migraine. For instance, *Felodipine* could be used as a proxy for hypertension [33], while *Clopidogrel* could be used as a proxy for blood clots and thromboembolisms [34]. *Felodipine* is a calcium-channel blocker used to treat hypertension by blocking calcium ions’ entry into the cell and minimising vascular smooth muscle contraction [33]. This mechanism has incremented calcium-channel blockers use as an off-label migraine medication [35]. In addition, clopidogrel is an antiplatelet drug commonly prescribed for thromboembolism prevention [36], and previous studies have demonstrated platelet aggregations to be significantly higher in individuals during a migraine episode [37, 38]. Therefore, our results suggest a potential vascular component to migraine, supporting the hypothesis in which vascular changes lead to migraine. We speculate that this relationship could be mediated by increased platelet clumping and vasoconstriction, perhaps as a consequence of hypertension and blood clots formation.

Lower socioeconomic status (SES) has been associated with a higher risk for chronic diseases [39, 40]. For instance, it has been noted that individuals with low SES are more likely to be exposed to hazardous substances at their workplace [39, 40]. In our study, self-reported migraine was a putative outcome of work environments in which individuals *work with paint, thinner or glues*, or are in a *workplace with a lot of diesel exhaust.* In contrast, other work environments, such as being in a *workplace often full of chemicals or other fumes*, did not show evidence for a potential causal association with self-reported migraine (Supplementary File 1). These results are consistent with previous studies suggesting that headaches and chronic neurological symptoms are prevalent among individuals exposed to mixture solvents and can be explained by the acute effect of solvents in the central nervous system [41, 42]. Similarly, it has also been reported that exposure to diesel exhaust particles is associated with neuroinflammation and headaches [43–45]. Although we cannot rule out the potential influence of SES-related variables such as educational attainment and assortative mating in the relationship between work environments and migraine. Our results are consistent with previous observational studies, suggesting that migraine could be an outcome of being exposed to diesel exhaust and working with solvents due to neuroinflammation and hazardous effects in the central nervous system.

The relationship between migraine and emotional distress has been described before; however, it has not been fully elucidated. For instance, several studies point out that migraine is associated with psychiatric disorders such as depression and anxiety [46, 47], while others suggest that the duration of a migraine episode is related to an impairment of anger control [48]. In the present study, experiencing the *death of a close relative in the last two years*, which is considered a major stressful life event that can lead to depression or anxiety [49], was potentially influencing an increase in self-reported migraine. In contrast, *ever feeling unenthusiastic / disinterested for a whole week* was identified as a putative consequence of self-reported migraine. Thus, our results show that stressful life events can be a risk factor for migraine, while migraine holds an impact on the psychological well-being of individuals, which in turn suggests that identifying psychological vulnerabilities among migraineurs is of great importance.

We note that statistical methods used in genetic epidemiology such as LCV and Mendelian randomisation test the potential causal effect of the genetic liability for a disease on the outcome [32]. Migraine can have its onset at around the age of 20, and some phenotypes identified to be causally associated with self-reported migraine can have a late-onset (i.e., stroke diagnosed by a doctor and neoplasms). Since the present study could not take into account the age of onset of migraine and other conditions, it is likely that genetic variants associated with late-onset phenotypes increase the risk for self-reported migraine, rather than the phenotype itself. Therefore, causal associations between self-reported migraine and late-onset phenotypes must be interpreted with caution.

We highlight the importance of triangulating results from multiple study designs, of which at least one should be an interventional study (i.e., a randomised controlled trial). However, interventional studies can be expensive, time-consuming, or unethical to perform. Therefore, identifying potential causal associations using statistical methods in genetic epidemiology could be the best option available. We suggest that the inferred causal relationships identified through LCV analyses should be testable hypotheses for future observational and genetic studies.

Important limitations of this study must be acknowledged. For instance, given that previous studies have highlighted racial and ethnic differences in migraine [50], and our analyses used participants of European ancestry, the generalisability of our results outside European ancestry individuals may be limited. Moreover, although more than 1500 traits were included in our analyses, causal associations with other traits may exist. Also, even though the LCV method uses aggregated information across the genome to increase statistical power, the GCP estimate still relies on the statistical power of GWAS [10, 12, 13], limiting the capability to infer causal effects for some traits. In addition, the presence of multiple latent factors could reduce the statistical power of the analysis and lower GCP estimates [10]. Lastly, since the bivariate nature of the LCV method aims to identify the predominant causal pathway between a pair of genetically correlated phenotypes [10], bidirectional causality between traits cannot be tested.

It is fundamental to consider potential biases in the design of the GWAS involved. Although the self-reported migraine sample used in the present study has been previously included in a large migraine meta-analysis, comprising ~ 52 % of the cases which allowed the identification of ~ 40 independent loci associated with migraine [15], we note that using self-reported data may have led to a recruitment bias since some patients may have misdiagnosed themselves with migraine [51]. Nonetheless, it has been reported that self-reported migraine can be congruent with a clinical diagnosis. Still, a self-report of migraine without a clinical diagnosis criteria is not able to delineate between subtypes such as migraine with or without an aura [52]. Similarly, we broadly define chronic headaches with the standard question of the International Headache Society [53] “Have you had headaches for more than three months?”, which could apply to primary or secondary types of headaches. As we mention in the methods section, since the focus of this study is migraine, we only used chronic headaches to boost statistical power for self-reported migraine due to the strong genetic correlation between both phenotypes (r_G_=0.83 s.e.=0.05).

Future studies should aim to assess whether the potential causal associations identified in the present study could be specific to a particular type of migraine and if they can be observed for secondary type headaches. As previous studies have noted [54], we suggest that medication use GWAS in this study should be interpreted as a proxy for the disease or disorder requiring the medication; however, as we mentioned earlier, due to the nature of self-reported data, the identification of migraine comorbidities only through medication use GWAS could be inaccurate and should be addressed with caution until confirmed by future studies. Related to this is the interpretation of medication use *Felodipine*, a proxy for hypertension, which could be indicative of a putative causal association between hypertension and secondary-type headache.

## Conclusions

We provide evidence for potential causal relationships between self-reported migraine and 1,504 different phenotypes. Our findings uncovered changes in blood vessels, particularly vasoconstriction and platelet clumping, potentially increasing migraine risk. Further, we show the putative influence of workplace environments increasing migraine risk and reveal emotional stress as a risk factor for migraine. Also, we reveal migraine’s potential impact on the psychological well-being of individuals. In addition, we raise the possibility of abdominal pain influencing a higher migraine risk. Altogether, our results confirm some causal associations contemplated in previous studies and point out testable hypotheses that, if confirmed, may open novel avenues in the understanding of migraine, which in turn can provide opportunities to improve the design of emerging treatments and drug targets.

## Supplementary Information

**Additional file 1: Supplementary File 1. **LCV output for migraine.

## Data Availability

Individual-level data for UK Biobank participants are available to eligible researchers through the UK Biobank (www.biobank.ac.uk). Individual-level data for 23andMe are available upon request through a Data Transfer Agreement and the appropriate application procedure (https://research.23andme.com/dataset-access/).

## References

[1] Genetics Home Reference Migraine. https://ghr.nlm.nih.gov/condition/migraine. Accessed 31 Jul 2020

[2] Victor TW, Hu X, Campbell JC (2010). Migraine prevalence by age and sex in the United States: a life-span study. Cephalalgia.

[3] Kikkeri NS, Nagalli S (2020) Migraine with Aura. In: StatPearls. Treasure Island, StatPearls Publishing 32119498

[4] GBD 2016 Headache Collaborators (2018). Global, regional, and national burden of migraine and tension-type headache, 1990–2016: a systematic analysis for the Global Burden of Disease Study 2016. Lancet Neurol.

[5] Weatherall MW (2015). The diagnosis and treatment of chronic migraine. Ther Adv Chronic Dis.

[6] Bahra A (2011). Primary Headache Disorders: Focus on Migraine. Rev Pain.

[7] Boran HE, Bolay H (2013). Pathophysiology of Migraine. Noro Psikiyatr Ars.

[8] Sutherland HG, Albury CL, Griffiths LR (2019). Advances in genetics of migraine. J Headache Pain.

[9] van den Maagdenberg AMJM, Nyholt DR, Anttila V (2019). Novel hypotheses emerging from GWAS in migraine?. J Headache Pain.

[10] O’Connor LJ, Price AL (2018). Distinguishing genetic correlation from causation across 52 diseases and complex traits. Nat Genet.

[11] Koellinger PD, de Vlaming R (2019). Mendelian randomization: the challenge of unobserved environmental confounds. Int J Epidemiol.

[12] Haworth S, Kho PF, Holgerson PL (2020). Assessment and visualization of phenome-wide causal relationships using genetic data: an application to dental caries and periodontitis. Eur J Hum Genet.

[13] García-Marín LM, Campos AI, Martin NG (2020). Inference of causal relationships between sleep-related traits and 1,527 phenotypes using genetic data. Sleep.

[14] García-Marín LM, Campos AI, Kho P-F (2021). Phenome-wide screening of GWAS data reveals the complex causal architecture of obesity. Hum Genet.

[15] Gormley P, Anttila V, Winsvold BS (2016). Meta-analysis of 375,000 individuals identifies 38 susceptibility loci for migraine. Nat Genet.

[16] Turley P, Walters RK, Maghzian O (2018). Multi-trait analysis of genome-wide association summary statistics using MTAG. Nat Genet.

[17] Bulik-Sullivan BK, Loh P-R, Finucane HK (2015). LD Score regression distinguishes confounding from polygenicity in genome-wide association studies. Nat Genet.

[18] Cuéllar-Partida G, Lundberg M, Kho PF et al (2019) Complex-Traits Genetics Virtual Lab: A community-driven web platform for post-GWAS analyses. bioRxiv 518027. 10.1101/518027

[19] Neale’s Lab (2018) GWAS Results. In: UK Biobank—Neale Lab. http://www.nealelab.is/uk-biobank

[20] Sodini SM, Kemper KE, Wray NR, Trzaskowski M (2018). Comparison of Genotypic and Phenotypic Correlations: Cheverud’s Conjecture in Humans. Genetics.

[21] Campos A, Kho PF, Vazquez-Prada KX et al (2020) Genetic susceptibility to pneumonia: A GWAS meta-analysis between UK Biobank and FinnGen. medRxiv 2020.06.22.20103556. 10.1101/2020.06.22.20103556 10.1017/thg.2021.27 34340725

[22] Noghani T, Rezaeizadeh M, Fazljoo H, Keshavarz SMB (2016). Gastrointestinal Headache; a Narrative Review. Emerg (Tehran).

[23] Cámara-Lemarroy CR, Rodriguez-Gutierrez R, Monreal-Robles R, Marfil-Rivera A (2016). Gastrointestinal disorders associated with migraine: A comprehensive review. World J Gastroenterol.

[24] Lankarani KB, Akbari M, Tabrizi R (2017). Association of Gastrointestinal Functional Disorders and Migraine Headache: a Population Base Study. Middle East J Dig Dis.

[25] Smith J, Parmely JD (2020) Ventral Hernia. In: StatPearls. Treasure Island, StatPearls Publishing 29763102

[26] Spellar K, Gupta N (2020) Diaphragmatic Hernia. In: StatPearls. Treasure Island, StatPearls Publishing 30725637

[27] Kavic MS (2005). Hernias as a source of abdominal pain: a matter of concern to general surgeons, gynecologists, and urologists. JSLS.

[28] Zuber M, Harder F (2001) Benign tumors of the colon and rectum. Evidence-Based and Problem-Oriented. Zuckschwerdt, In: Surgical Treatment

[29] Mason BN, Russo AF (2018). Vascular Contributions to Migraine: Time to Revisit?. Front Cell Neurosci.

[30] Jacobs B, Dussor G (2016). Neurovascular contributions to migraine: Moving beyond vasodilation. Neuroscience.

[31] Bigal ME, Kurth T, Hu H (2009). Migraine and cardiovascular disease: possible mechanisms of interaction. Neurology.

[32] Siewert KM, Klarin D, Damrauer SM (2020). Cross-trait analyses with migraine reveal widespread pleiotropy and suggest a vascular component to migraine headache. Int J Epidemiol.

[33] Bansal AB, Khandelwal G (2019) Felodipine. In: StatPearls. Treasure Island, StatPearls Publishing

[34] Fisch AS, Perry CG, Stephens SH (2013). Pharmacogenomics of anti-platelet and anti-coagulation therapy. Curr Cardiol Rep.

[35] Calcium Channel Blockers (2017) In: LiverTox: Clinical and Research Information on Drug-Induced Liver Injury. National Institute of Diabetes and Digestive and Kidney Diseases, Bethesda (MD). https://www.ncbi.nlm.nih.gov/books/NBK548577/

[36] Beavers CJ, Naqvi IA (2020) Clopidogrel. In: StatPearls. Treasure Island, StatPearls Publishing

[37] Sarchielli P, Gallai V (2001). Platelets in migraine. J Headache Pain.

[38] Hanington E (1981). Migraine: a platelet disorder. Lancet.

[39] Clougherty JE, Souza K, Cullen MR (2010). Work and its role in shaping the social gradient in health. Ann N Y Acad Sci.

[40] Burgard SA, Lin KY (2013) Bad Jobs, Bad Health? How Work and Working Conditions Contribute to Health Disparities. Am Behav Sci 57. 10.1177/0002764213487347 10.1177/0002764213487347 PMC3813007 24187340

[41] Dick FD (2006). Solvent neurotoxicity. Occup Environ Med.

[42] Wang JD, Chen JD (1993). Acute and chronic neurological symptoms among paint workers exposed to mixtures of organic solvents. Environ Res.

[43] Kim BG, Lee PH, Lee SH (2016). Long-Term Effects of Diesel Exhaust Particles on Airway Inflammation and Remodeling in a Mouse Model. Allergy Asthma Immunol Res.

[44] Dales RE, Cakmak S, Vidal CB (2009). Air pollution and hospitalization for headache in Chile. Am J Epidemiol.

[45] Gerlofs-Nijland ME, van Berlo D, Cassee FR (2010). Effect of prolonged exposure to diesel engine exhaust on proinflammatory markers in different regions of the rat brain. Part Fibre Toxicol.

[46] Marcus DA (2000). Identification of patients with headache at risk of psychological distress. Headache.

[47] Peres MFP, Mercante JPP, Tobo PR (2017). Anxiety and depression symptoms and migraine: a symptom-based approach research. J Headache Pain.

[48] Perozzo P, Savi L, Castelli L (2005). Anger and emotional distress in patients with migraine and tension-type headache. J Headache Pain.

[49] Keyes KM, Pratt C, Galea S (2014). The burden of loss: unexpected death of a loved one and psychiatric disorders across the life course in a national study. Am J Psychiatry.

[50] Nicholson RA, Rooney M, Vo K (2006). Migraine care among different ethnicities: do disparities exist?. Headache.

[51] Gebhardt M, Kropp P, Hoffmann F, Zettl UK (2019). Headache in the course of multiple sclerosis: a prospective study. J Neural Transm.

[52] Wang J, Zhang B, Shen C (2017). Headache symptoms from migraine patients with and without aura through structure-validated self-reports. BMC Neurol.

[53] Murphy C, Hameed S (2021) Chronic Headaches. In: StatPearls. Treasure Island, StatPearls Publishing 32644509

[54] Wu Y, Byrne EM, Zheng Z (2019). Genome-wide association study of medication-use and associated disease in the UK Biobank. Nat Commun.

